# A Distributed Signature Detection Method for Detecting Intrusions in Sensor Systems

**DOI:** 10.3390/s130403998

**Published:** 2013-03-25

**Authors:** Ilkyu Kim, Doohwan Oh, Myung Kuk Yoon, Kyueun Yi, Won Woo Ro

**Affiliations:** 1 School of Electrical and Electronic Engineering, Yonsei University, Seoul 120-749, Korea; E-Mails: ilkyu.kim19@gmail.com (I.K.); ohdooh@yonsei.ac.kr (D.O.); myungkuk.yoon@yonsei.ac.kr (M.K.Y); 2 Software Platform R&D Laboratory, LG Electronics Inc., Seoul 137-130, Korea; E-Mail: kyueun@ieee.org

**Keywords:** network intrusion detection system, multiple pattern matching, distributed computing, Wu-Manber, Snort

## Abstract

Sensor nodes in wireless sensor networks are easily exposed to open and unprotected regions. A security solution is strongly recommended to prevent networks against malicious attacks. Although many intrusion detection systems have been developed, most systems are difficult to implement for the sensor nodes owing to limited computation resources. To address this problem, we develop a novel distributed network intrusion detection system based on the Wu–Manber algorithm. In the proposed system, the algorithm is divided into two steps; the first step is dedicated to a sensor node, and the second step is assigned to a base station. In addition, the first step is modified to achieve efficient performance under limited computation resources. We conduct evaluations with random string sets and actual intrusion signatures to show the performance improvement of the proposed method. The proposed method achieves a speedup factor of 25.96 and reduces 43.94% of packet transmissions to the base station compared with the previously proposed method. The system achieves efficient utilization of the sensor nodes and provides a structural basis of cooperative systems among the sensors.

## 1. Introduction

Sensor nodes are widely used in various applications such as air pollution monitoring, forest fire detection, and anti-theft system. [1]. The advances in the technology of micro-scale electronic devices have enabled the development of tiny sensors that are inexpensive, consume low power, and multifunctional [2]. In addition, the improvement of the wireless communications technology has provided an untethered data communication protocol over a short distance. These technologies enable a number of sensor nodes to perceive and collect data from the external environment. Wireless sensor networks (WSNs) are utilized for various monitoring work including the long-term monitoring of harsh environments; thus the nodes should be sustained for a long time with a limited battery and respond well to external changes [3,4].

However, the WSN is vulnerable to many malicious attacks called network intrusions. This is because the sensor nodes utilize wireless communications and are deployed in open environments such as mountainous and urban terrains. There is a possibility of obtaining physical access to the sensor networks and intercepting the data from the networks [5]. At worst, the intrusions could be extremely dangerous because the WSN that monitors chemical gases or battlefields could be malfunctioning [6].

Managing a WSN is demanding work because of network intrusions, especially denial-of-service (DoS) attacks [6]. Intrusion detection systems can be easily made ineffective because the attacks contain messages that are similar to regular client requests. Many DoS attacks exist in each network layer, and resources are rapidly exhausted, disrupted, or even destructed by these simple and repeated attacks [6]. Furthermore, networks are easily exposed to the attacks because the networks normally deal with too many nodes to be controlled independently, and each node has restricted hardware resources to prevent the attacks [7,8]. Consequently, each compromised sensor node might accidentally deliver harmful data to the central server when the node is compromised by malicious patterns. To prevent attacks, efficient security solutions for the sensor nodes are needed to maintain the reliability of the networks.

Network intrusion detection system (NIDS) has been proposed to prevent the sensor networks from network-based malicious attacks. Each attack is analyzed as one of the many malicious signatures (patterns) and summarized by pattern data groups. From the pattern groups, the system avoids damage from the same or similar attacks. Using a pattern-matching algorithm that uses the intrusion pattern sets, the system can analyze incoming packets and filter out malicious attacks [1,3,9]. However, few studies on pattern-matching algorithms for sensor nodes have been conducted because most sensor nodes have constrained resources for low power.

In this paper, we propose a novel security solution for sensor networks by modifying the traditional Wu–Manber (WM) pattern-matching algorithm. Because the sensor node has limited performance, memory size, and power, a full implementation of a general pattern-matching algorithm on each node is difficult. To solve this issue, the algorithm is divided into two major steps: a shifting step and an exact pattern-matching step. The two steps are allocated respectively to the sensor node and the base station according to the demands of memory allocation and computing power. The modified shifting step is optimized for limited resources and is executed by the sensor node. The base station in the cluster-based structure is authorized to execute exact matching, which is difficult to process on the sensor node. Transmitted packets with searched results for each sensor node are processed by the base station. By answering each node with the result, cooperative pattern matching can be achieved.

We represent a framework between the sensor node and base station that can perform distributed intrusion detection with the WM algorithm. We conduct evaluations of the framework and analyze the results. The results show that the framework achieves a performance improvement for both the sensor node and the base station. The results allow the base station to process a large number of sensor nodes more efficiently.

The rest of this paper is organized as follows. First, we introduce background information about the various methods of detecting network intrusions in sensor nodes and related work in Section 2. In Section 3, the framework for network intrusion detection is introduced. Then, we explain the proposed algorithm in Section 4, which is a cooperative WM algorithm to develop the resources of both the sensor node and the base station. We evaluate the performance of the work in Section 5. Finally, conclusions with plans for future work are discussed in Section 6.

## 2. Background

This section introduces an NIDS that is developed to detect or prevent various network intrusions. The WM pattern-matching algorithms used to detect intrusion signatures are also introduced.

### 2.1. Network Intrusion Detection System

Network intrusions refer to malicious attacks such as attempting DoS attacks, intercepting packet payloads, and cracking target nodes. These intrusions are detected and prevented by a security technology called intrusion detection [10]. NIDS is a complete system equipped with the intrusion detection technology. The system consists of all devices and information about the networks, such as host, routers, and monitoring results [1].

Detecting intrusions such as DoS is difficult to implement because most intrusions pretend that they are general packets. Moreover, many intrusions have polynomial characteristics and are not easily found by comparison with specific patterns. The implementation of an NIDS is difficult because the detection algorithm should not disturb its own intended flow of sensing work.

The intrusion detection systems are classified into misuse detection and anomaly detection systems [3,11]. A misuse detection system uses pattern-matching algorithms with predefined patterns called intrusion signatures. Most of the signatures were obtained by an empirical method. In other words, the signatures are created by obtaining the common context of payloads that are revealed as attacks. Firmly constructed databases of these signatures can prevent network devices from the same attacks again. However, this algorithm is unsuitable for the detection of newly introduced or polymorphic intrusions because only known patterns can be detected. A heuristic matching method that allows wild-card characters is used to supplement misuse detection [12].

Implementing a misuse detection system in sensor nodes has been studied in several previous works. Because the nodes have limited resources, many researchers introduced outlier detection systems that deploy additional distributed monitoring nodes containing larger resources than the others [8,13]. The outlier nodes only conduct intrusion detection work, and deliver the detection result to the base station and other sensor nodes. Amin *et al.* proposed a robust intrusion detection system (RIDES) that unified detection schemes, including the signature-based detecting system and anomaly detecting system [14]. The algorithm converted a packet's payloads to signature codes based on Bloom filters. Baig introduced five phases for detecting intrusions, including pattern recognition and feedback of the results [4].

An anomaly detection system recognizes normal activities in the networks. Comparing received packets with these activities, malicious intruders can be detected by the system. The system can find deliberately modified or unknown signatures that are difficult to detect by misuse detection systems. However, it is still difficult and time-consuming to determine a profile of the normal activities [7]. The profile is determined by input data, output results, data types, and labels. Stochastic approaches are generally used to set the profile [15].

Anomaly detection systems have also been regarded as one of the many intrusion detection systems. Distributed intrusion detection systems (IDSs) that have a systematic anomaly-detecting process were developed [16,17]. Similar to outlier systems, additional agents such as gateways are dedicated to execute the intrusion detection schemes. An intrusion alert function based on anomaly detection was also introduced [11]. The system can validate signals among the sensor networks using these functions. In addition, packet traffic can be a criterion for detecting an anomaly in networks. A matching algorithm, which regards observed packet traffics as specified patterns, was also proposed [15,18].

Snort is one of the well-known IDSs based on a hybrid algorithm that uses both misuse detection and anomaly-based inspection [19]. Snort has rule sets that define malicious attacks. Each rule contains information about a protocol, port number, and context of the packet. The rule sets support regular expressions that contain wild-card characters, and the sets require the algorithm to perform both signature and heuristic matching. Many researchers have conducted studies to accelerate and optimize the rule set finding mechanisms such as pattern-matching algorithms [9,20,21]. However, these efforts are still difficult to implement in sensor networks because the schemes should be modified for the constrained resources of the sensor node.

### 2.2. Multiple Pattern Matching Algorithm

A string-matching algorithm is a process of searching string subsets in a specific sentence called a text. The algorithm is used for finding words in a web page or a textbook, DNA pattern recognition, and network intrusion detection. This classical algorithm has issues of both memory efficiency and performance.

The WM algorithm is a powerful pattern-matching algorithm that can detect multiple patterns simultaneously [22]. This algorithm is one of the classical multiple pattern matching algorithms improved from the Boyer–Moore algorithm [23]. In addition, the WM algorithm is a shift-or algorithm using a hash table and prefix table that seeks a substring of the text to determine the shift amount. The substring, normally called a block, has two or three sequential characters [22]. These characters form the criterion for the shift amount. Referring to the hash- (*i.e.*, the shift) table indexes, the algorithm obtains the shift amount from the table entries. Zero-shifting entries mean that the substring is a suffix of the pattern sets and have a chance to be matched with the patterns. The prefix table gives additional information about prefix subsets of the patterns to match all characters. In this way, the algorithm can skip two or more characters in the text. The skipping method enables the algorithm to find multiple patterns at once and increases the searching speed. Snort uses a modified version of Wu–Manber [24].

**Figure 1.**
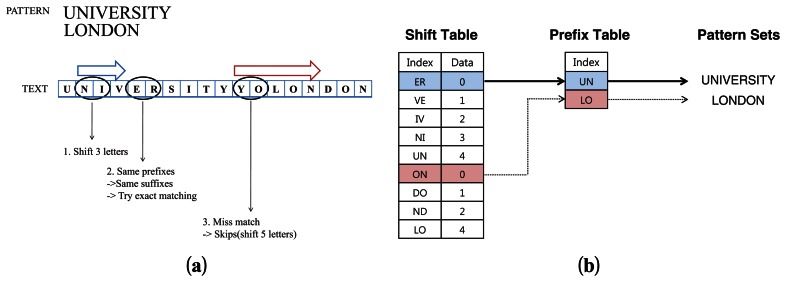
Shifting examples using the WM algorithm. (**a**) The shifting processes; (**b**) The two tables of WM.

Figure 1(a) shows a simple example for finding patterns using the WM algorithm. Let us first assume that there are only two patterns to search: “UNIVERSITY” and “LONDON”. The WM algorithm first constructs the shift and hash tables from the patterns before it starts the actual matching operation. Because the shortest pattern “LONDON” has six characters, the minimum length *m* is set as 6. To make an appropriate shift table among *m* characters, the pattern “UNIVERSITY” is also considered as the first six letters “UNIVER.” Assuming that the block size *B* is 2, we can make the shift table based on the two patterns. For example, the subsets “ER” and “ON” are zero-shifting entries and “VE” and “DO” indicate the shift value as 1. The pointer of the text string is continuously shifted by the entries of the shift table until the pointer reaches the end of the text string.

Figure 1(b) shows both the suffix and prefix tables established by the two patterns in Figure 1(a). The algorithm first looks at block “NI” of the text, and the shift table indicates the algorithm shifts three characters. Then, the shift value of the next block “ER” is zero. In this case, the algorithm returns to *m* − *B* = 4 characters and takes another block of that position called the prefix block. The prefix block “UN” is also matched with the prefix table. Therefore, the algorithm starts to compare the text with the entire pattern and finds that the string matches one of the given patterns. The other case is shift-table mismatching. When mismatching occurs, the shift table indicates a full shift that can be derived by *m* and *B* as *m* − *B* + 1 characters. In this figure, the block should be shifted to five characters. Then, the block (*i.e.*, “YO”) also leads to shift five characters. For the next case, the pointer indicates “DO” and eventually finds out the pattern “LONDON”.

The patterns are always detected, although the algorithm skips two or more characters. For example, the third case (*i.e.*, “YO”) in the Figure 1(a) leads to shift five characters. For the next case, the pointer indicates “DO” and eventually finds out the pattern “LONDON”. Because the amount of the full shift is restricted by *m*, the pointer never misses the directly followed patterns. However, the algorithm improperly finds short-sized pattern sets for the same reason.

## 3. Sensor Node-Based NIDS Frameworks

In this section, we introduce an NIDS framework modified for small-sized sensor nodes. This section first introduces the computation offloading method from a resource-constrained sensor node to a resource-rich base station. This section also describes MinWM (Minimized Wu–Manber algorithm) optimized for each device. MinWM uses a process dividing scheme and makes two nodes to manage the shift table and prefix table independently. Packet transmission management among the network is also discussed briefly where sensor nodes determine whether a packet is a malicious attack or not.

### 3.1. Networked Sensor Platform

The main objective of the node is to deliver gathered sensing information such as light, temperature, radio frequency, and vibration to central hosts called base stations. The base station creates valuable information from the gathered data. In this way, we can apply a cluster-based sensor system to observe large-scale natural phenomena and develop unmanned management systems of specific regions (e.g., industrial management and urban air-pollution observation).

The sensor nodes are low-power and low-performance devices because they are not connected with external wires and operate on internal battery power. Generally, a node has 64 KB memory and a micro-controller below 16 MHz frequency. Therefore, only light-weighted process that utilizes minimal resources can be implemented on the devices. Moreover, the detecting process should not disrupt the periodic sensing works (*i.e.*, the process should be terminated in a short time). Highly time and resource demanding process cannot be executed on the sensor nodes.

The framework based on MinWM is similar to other cooperative NIDS [8,14]. Both the nodes and the base stations take different jobs according to their hardware resources. The objective is to improve the overall performance of the NIDS and to decrease unnecessary network transmission. As we mentioned, the sensor node should not perform entire signature matching. Instead, the pattern matching algorithm should be modified to reduce resource usage. The strategy of MinWM is to divide the WM algorithm into table-matching and actual-matching steps. This approach allows the node to implement WM-based detection systems and utilizes the base station to boost the throughput of the entire detection process.

**Figure 2.**
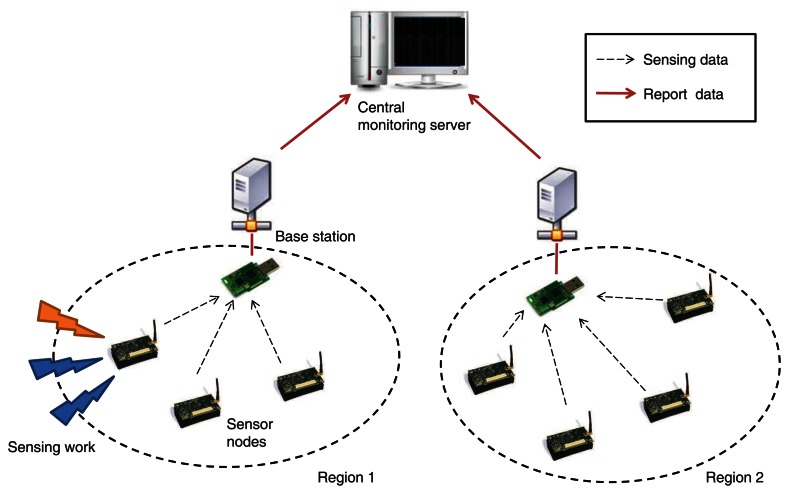
The system model of the networked sensor platform.

Figure 2 shows an example of the system model based on the multiple networked sensors. Each node is assigned to certain regions and gathers external sensing information for predefined purposes. For a certain time period, the nodes have transmitted the data to the assigned base station for summarization of the sensing works. Meanwhile, the base station of a particular region is responsible for collecting data and managing sensors in the same region. The packet from each base station to a top monitoring server represents the summarized sensing data of their own regions. Because of this cluster-based structure, the general analysis at the top monitoring server is simplified by these base stations.

**Figure 3.**
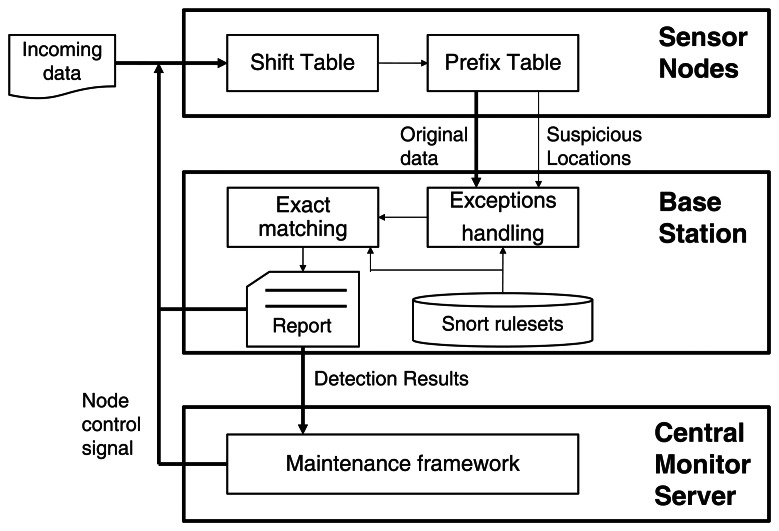
The division of processes of the distributedWM algorithm.

The intrusion detection systems based on the Snort signature-set checks additional information of incoming packets such as port numbers and protocol types besides the payloads. In other words, the system only inspects the payload of an incoming packet when the packet comes through a specific port number and protocol type corresponding to the signatures. The packet's payload is checked by both the shift table and the prefix table before transmitting the sensing data. The result of the inspection is inserted into the original payload. The base station is responsible for the remaining detection work (*i.e.*, the exact matching). Using the additional data from the sensor node, the attack attempts can be quickly determined by the base station, which has more computing resources than the sensor nodes. The sensor nodes and base stations notify other nodes of an intrusion if the packet is revealed as a malicious attack. Each sensor node has the same algorithm for consistent intrusion detection. Figure 3 shows how the general WM steps are divided and performed by the sensor nodes and the base stations.

To process the work efficiently, MinWM is based on a sensing work provided by TinyOS's library. The overall process of the system is shown in Figure 3. The sensor nodes transmit the packet that contains the original data and locations of suspicious attacks. The base station operates the actual matching schemes using the location data set by each node. The result is also transmitted to the sensor nodes that suspect attacks. The informing step is a simple broadcasting step; however, other traceback mechanisms can be applied to improve network safety [25,26].

### 3.2. Intrusion Detection Schemes

The sensor node checks the existence of intrusions within the time period. Comparing substrings of the payload with shift-table entries, the node can examine substrings with high speed. We focus on the DoS rules among the Snort signatures because being exposed to DoS attacks can lead to a dangerous situation for the WSNs [6]. The table that was created by the content fields of the rules contains information about the amount of shifts. The skipped characters are irrelevant to the matching suffixes [27]. The shifting also indicates that suffix matching may exist at the shifting point. The algorithm searches the prefix table only if the shift table indicates a zero-valued entry. This skipping method helps the sensor node to finish searching the context in time despite its limited resources.

The prefix table represents the string's prefix portions of the NIDS content source. The prefix table rechecks a suspicious payload that indicates zero entries. In general, the exact-matching step occurs when the signature satisfies the conditions of the two tables. The second table can reduce incidences of warning alert messages and increases the matching accuracy notably.

The sensor node does not contain entire signatures owing to two reasons. The first reason is that the exact-matching step requires a large portion of the WM algorithm's execution time. The substrings are compared with all characters in the signatures that have the same suffixes and prefixes. Considering the extremely constrained computation power, this process is too slow to be executed while maintaining the periodic sensing process. Secondly, the sensor node does not have sufficient space to keep entire signatures. A low-end sensor node is known to be unsuitable to deal with general patterns without modifying the signatures [28]. Instead of processing all signatures on the sensor node, we mark the location of a suspicious string as a bookmark and pass it to be processed by the base station. Only one byte in size is sufficient to represent a bookmark in the packet because the general packet size is less than 128 bytes. The bookmark can also represent both suffix and prefix information because the block size and minimum length of the signatures are defined in advance.

The base station receives packets that contain the inserted location information (*i.e.*, the bookmarks) from multiple nodes. Using the bookmarks, MinWM checks suspicious signatures marked by the sensor nodes to determine whether the packets are actual attacks or not. The suffix and prefix information substrings are sent to the base station in the form of bookmarks. This approach reduces the time to find matching candidates. In addition, MinWM also skips the step where the suffix and prefix tables are compared, which is already executed on the sensor nodes. As a result, the MinWM framework can achieve higher throughput than processing the entire WM algorithm at the base station. A more powerful aspect is that MinWM can even skip the whole-detection steps if the payload checked by the node does not contain any bookmarks. Therefore, reducing the load of the base station helps in processing a large number of sensor nodes efficiently. While the prefix and suffix tables are simply utilized for each sensor node, the base station only focuses on complete signatures. In this way, both the sensor nodes and the base stations can achieve the objectives of memory efficiency and performance.

### 3.3. Packet Transmission Management

The detection results of the packet are sent to the sensor nodes that previously transmitted the packet. Considering the broadcast capabilities of the sensor nodes, a traceback for the network intrusion is emphasized. The sensor nodes that transmit the intrusions are distinguished and managed by the intrusion system according to the policy of the system. Traceback mechanisms have been studied by other researchers [25,26]. A small portion of trace information is inserted into the node, and the system is prepared to trace the information using the bloom filter, although a simple traceback mechanism is assumed in the MinWM framework.

**Figure 4.**
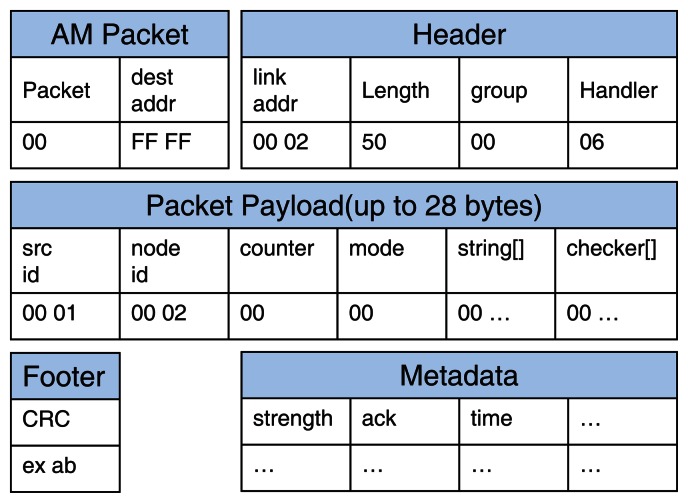
The packet format for the wireless sensor network.

Figure 4 shows the message format of MinWM. This is the format of a TinyOS 2.x message and ZigBee protocol [19]. The format mainly consists of five categories: AM packet, header, packet payload, footer, and metadata. The AM packet represents the category of the packet and its destination nodes. The header field contains the overall information about a packet, such as the source address, length, and packet groups. The footer field has cyclic-redundancy-checking (CRC) parity data to verify the contents of the packet. The metadata field contains additional information for the networks. The “dest addr” in the AM packet contains the address of the node that should receive the packet. For example, the address “FF FF” indicates a broadcasting mode, which means that the packet is transmitted to all nearby nodes.

**Figure 5.**
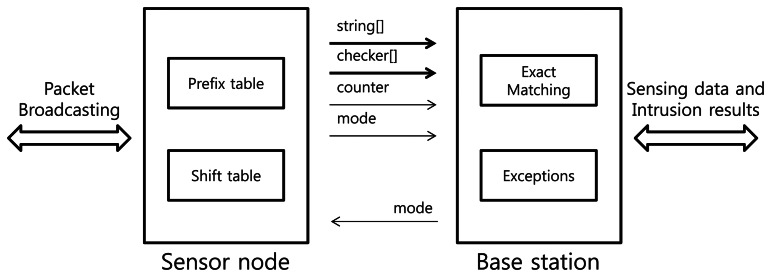
The packet messages between sensor nodes and a base station.

Figure 5 indicates the overall message transmissions to conduct pattern matching between the sensor nodes and the base station. The payload data is transmitted by the encapsulated packet. Although the general WM system needs only the data field “string” in the packet payload to process the sensing data, additional fields that occupy small payload sizes are added. As we discussed in Sections 2.2 and 3.2, the sensor node compares the string field with the suffix and prefix tables. If a suspicious substring is detected, the position of the suffix is written on the checker array, and the total number of intrusion candidates is written on the counter field. The base station examines the partial substrings of the packet's payload using the bookmark data. The size of the packet is sufficient enough to contain the two additional fields because the ZigBee protocol provides a bandwidth of up to 20 KBps [2]. In addition, the data is compatible with other systems because the system uses general packet structures with minor conversions.

Distributed sensor nodes and the centralized base station notify each other of the classification of the transmitted packet. In other words, the packet of the sensor nodes contains additional information about checking status, including warnings of the intrusions. The packet of the base station has the results of intrusion detection and actual alerts of intrusions. An additional field “mode” makes each node recognize the status of incoming packets. After each detection step is finished, the status is determined and written at the one-byte field data. The field is also used for the network management methodology consisting of two or more sensor nodes. According to the network policy, each node and base station can notify each other of the current detection status of the packet. Because the field represents a total of 256 different states, the field can be used for the MinWM framework with a large number of sensors.

## 4. Modified Tables of the Wu–Manber Algorithm

Each sensor node commonly has two tables created by substrings of intrusion signatures. However, the tables based on the original WM algorithm cannot be used owing to two limitations: the structure of Snort signatures and restricted resources of the sensor nodes. In this section, we discuss the problems and novel solutions for the sensor nodes and the base station. We first minimize the shift and prefix tables to allocate them into the memory of the sensor node. Each entry of the tables is classified by single characters to reduce the required memory size. In addition, we prepare an additional scheme to deal with specific patterns that are difficult to implement.

### 4.1. One-Character Classification

Snort has recommended using the WM algorithm to find intrusions because it has an advantage in memory efficiency as compared with other pattern-matching algorithms [19]. However, the memory requirements of the algorithm are still too large to reside in the memory of the sensor node. Generally, the suffix and prefix tables of the WM algorithm use combinations of multiple characters to allocate the addresses of each entry. The address is derived from arithmetic equations of the characters as a block. The optimal *B* is known to be either *B* = 2 or *B* = 3 [22]. The system requires an additional one byte of data for every entry to represent a total of *C* = 256 different expressions. Although we set *B* = 2, the shift table requires the memory size to be *C^B^* × 1 = 64 KB. Considering that almost all sensor nodes have a memory size of less than 64 KB, the table cannot be allocated without modification.

Instead of using multiple character combinations, we propose tables based on single characters, i.e., *B* = 1. In this case, the required memory size of each table is *C^B^* ×1 = 256 bytes, which is significantly smaller than the memory size using multiple character combinations. However, the reduction of block size can cause significant performance degradation because the shift amount is also decreased by the block entries. A smaller table size contains a smaller resolution for detection clues. In other words, the suffix table has only 256 combinations of shifting information if the table consists of single character entries. As a result, the shift entry is forced to have smaller shifting amount. Still, this table-based approach shows better throughput than other algorithms based on single-character comparison owing to the skip method.

The prefix table can contain many more entries than the shift table can. The prefix table requires 256^2^ = 65536 = 64 KB memory size at maximum if all two-character combinations appear among the signatures. However, allocation of the maximum memory size is not always necessary. The required memory size strictly depends on the number of signatures. As we mentioned in Section 2.2, MinWM examines the prefix table only if the suffix table indicates a zero shift. In addition, the prefix table can be represented by linked lists of prefix substrings indicated by a pointer of the suffix entry. In this case, the memory requirements of the prefix tables depend on the number of prefix characters in the signatures. In contrast to the original WM algorithm, MinWM on the sensor nodes does not make a copy of the matching candidates. MinWM simply records the positions of matching in the payload context to reduce the size of the packet and provide efficient control for the exact matching executed on the base station.

The performance and memory requirements heavily depend on the number of signatures. Therefore, a small number of packets are used to achieve high throughput. DoS signatures are suited for this scheme because the number of rules is less than 100. Considering overlapped signatures in the rules, this structure can allow the sensor node to provide a partial intrusion detection scheme with minuscule resource consumption.

### 4.2. Short String Exceptions

The content fields of each rule are considered as the signatures. Because the length of the fields is variable, generation of signature sets for all rules is difficult. Strings less than two characters long significantly reduce the performance of the WM-based algorithm because the maximum shifting value depends on *m* and *B* (*i.e.*, *m* − *B* + 1). Only one signature that has one character prevents shifting by more than one character.

To solve the problem, MinWM uses the structure of the Snort rules. In fact, the content fields shorter than two-character strings provide additional position information to clarify the ambiguous strings. Moreover, the position indicates either the exact or near position of the first letter in the string. Instead of inserting the short signatures into the two tables, we make exceptions for the signatures. The additional step is prepared to account for the exceptions before the algorithm executes the exact-matching step. In the additional step, the algorithm verifies the exceptions at the string locations specified by the rules. MinWM simply checks bytes in the location described in the exceptions and does not execute the whole string-finding algorithm for the short patterns. Consequently, MinWM requires almost no additional resource and execution time. In addition, the signature sets without short patterns create small-sized prefix tables and a shift table with the large shifts.

## 5. Evaluations

In this section, we only consider networks that consist of a single sensor node and a base station because MinWM only considers implementing the distributed WM algorithm among two nodes. Although the evaluations are conducted with two nodes, MinWM can be expanded to multiple-node-based networks owing to the simplicity of the system. However, the networked system should control the overall traffic transmitted by the nodes. Traffic management is already discussed in Section 3.3. We first explain the environment of the sensor nodes and then discuss the results in detail.

### 5.1. Experimental Environments

We conduct evaluations using a Kmote device, which is a typical low-end sensor node. The device has an 8 MHz MSP430 microcontroller and a CC2420 radio chip that is compatible with other IEEE 802.15.4 (*i.e.*, ZigBee) based devices. The microcontroller has 10 KB RAM and 48 KB flash memory. If the size of each entry is set to 1 byte, the shift table requires only 256 bytes, and the table is sufficiently allocated in the device. The size of the prefix table depends on the number of signatures in a node. Considering the number of DoS signatures in Snort, the flash memory size sufficiently contains the prefix table.

In general, the misuse detection systems consider payload contents in target packets as strings. This is due to that most malicious rules are defined in a form of strings and traditional string matching algorithms can be effectively utilized to detect malicious data. Implementing a misuse detection system in sensor nodes also applies this approach [4,14,18]. In fact, the MinWM framework also detects intrusion signatures on the strings, which represent the payloads of incoming packets.

The MinWM framework uses a rule-based intrusion detection scheme, and Snort v2.9 DoS rule sets are used for the target signatures. A total of 77 rules are selected and contained in the sensor nodes to detect specific attacks. Generally, one rule in Snort consists of one or more string sets. The rule also has information about the distances between two sets. MinWM considers each string set as a single pattern. If one or more patterns are matched, the locations are recorded in the bookmarks and inserted into the sending packet. From the packet, the original context and the added bookmark can be delivered to the base station. Because the base station knows the locations of the suspicious strings, pattern sets that contain wildcard characters are quickly determined by checking the distances of the matching patterns. Strings below two characters are considered as exceptions, as described in Section 4.2, and are processed by the base station before exact matching.

The base station processes not only the exceptions but also other regular expressions that are not easily implemented on the sensor nodes. Specific signatures in Snort are written in a Perl-compatible regular expression syntax (PCRE) [29]. The signatures can be searched by the base station because the base station has the resources to load the PCRE library. A queue structure is prepared to receive multiple packets, and the structure helps the base station to use any parallelization techniques such as multi-threaded computing.

### 5.2. Performance Improvement of the Distributed Detection System

The time complexity of the detection scheme is derived by the original author of the WM algorithm. The throughput of the algorithm is dependent on *B*, the total length of the text *N*, and *m* [22]. MinWM needs time to construct the shift and prefix tables; however, we do not consider this in this evaluation because the tables are predefined and loaded in the memory. Including the time to compute hash functions *O*(*B*), the complexity can be given by *O*(*BN*/*m*) [22]. RIDES, which uses bloom filters to find signature codes, is presented for comparison with the proposed algorithm. RIDES represents a complexity of *O*(*N* + *∊*), where *∊* is the number of patterns [14]. As we mentioned in Section 4.1, the minimized block size *B* = 1 reduces the load to compute hash functions and increases the overall throughput. Considering another parameter set with *m* = 3, the algorithm theoretically shows better throughput than RIDES.

**Figure 6.**
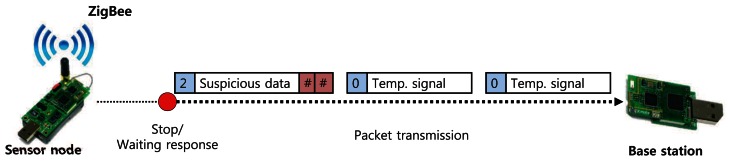
An intrusion emulation on the proposed sensor network.

Simple attack emulations are conducted on the sensor nodes as shown in Figure 6. A target node periodically sends the current temperature to the base station; the time interval is set as one second in this evaluation, but it can be changed. We artificially generate attack signals that contain DDoS signatures and insert them into the sensor node through wireless networks. A sensor node that receives these signatures can detect them and make an alert signal. In the figure, only a certain part of payload in the alert signal is presented: counter, suspicious data, and checker referred in Figure 4. After the alert signal is sent to the base station, the node stops sensing the current temperature and waits for response from the base station. The base station verifies the suspicious data on the alert signal and then reports the result by sending the response signal. Under this emulation environment, we evaluate performance of the proposed approach.

Table 1 indicates the elapsed time and power consumption of the two algorithms in the sensor node. We have evaluated each test for 1,000 times and the average is represented in the table. We find that MinWM detects DoS intrusions more efficiently than RIDES because of the structural advantages of the approach. To find the number of intrusions, MinWM uses shifting techniques that can skip one or more characters, while RIDES uses hash techniques derived by the Rabin–Karp algorithm [30]. RIDES is known to be suitable for finding large numbers of patterns because the algorithm simplifies the exact-matching steps instead of applying the character-shifting schemes. In Table 1, the searching speed of RIDES remains constant while the searching speed of MinWM decreases according to the number of the bookmarks.

The power consumption in Table 1 is evaluated from the measured time and the power consumption of the sensor node [31]. The sensor node that integrates MinWM consumes 1.51 *μWs* to detect any DoS attacks on a single packet when the number of bookmarks is zero. On the other hand, when using RIDES to detect DoS attacks, the sensor node requires 128.08 *μWs* energy. As a result, RIDES consumes up to 126.57 *μWs* more energy for inspecting a single packet compared with MinWM.

**Figure 7.**
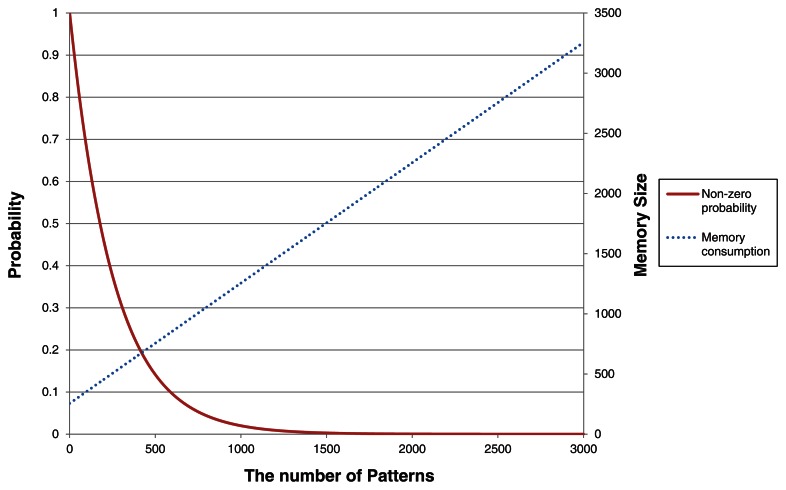
The non-zero probability and required memory occupation for the patterns.

Using a simple probabilistic approach, we evaluate the performance of MinWM. Setting Σ to be the number of alphabets, a matching probability of only one pattern that equals the probability of zero shifting is simply derived as (1/Σ*^B^*). In addition, no pattern is matched with a substring of the text if actual shifting would occur. From the derivation, the non-zero shifting probability equals (1 − 1/Σ*^B^*)*^∊^*. This equation shows that the performance is strongly related to the number of patterns to be detected. Figure 7 reveals the relationship between the signature size and the non-zero probability. In the proposed approach, each signature only requires one additional byte in the prefix table. Although the memory is sufficient to contain all signatures in Snort, the general WM algorithm restricts the appropriate number of patterns. Only 500 signatures drop the non-zero probability below 20% and definitely decrease the throughput of the algorithm. However, MinWM is still considered a reasonable method because the DoS attack signatures are limited in number and overlap with each other. Moreover, the problems of detecting a large number of patterns are solved by the distributed processing of multiple sensor nodes.

To verify the effect of the bookmarks, we have performed simple evaluations. A number of strings are prepared and inserted into the process of the sensor node. As a result, the strings contain information about the location of suspicious substrings (the bookmarks). The actual matching step is executed using the bookmarks, and the throughput of the base station is calculated by measuring the processing time. To show the improvement, we measured the throughput of the general WM algorithm with the same strings. The evaluations are conducted on an identical machine equipped with an AMD Phenom II X4 955 processor running at 3.2 GHz and equipped with 4 GB of DDR3 RAM. The source patterns are string sets that are generated randomly with a length of 50. The number of patterns is varied to observe the performance of processing incessant data inflow. The throughput of two methods is calculated by the processing time and total string sizes.

**Figure 8.**
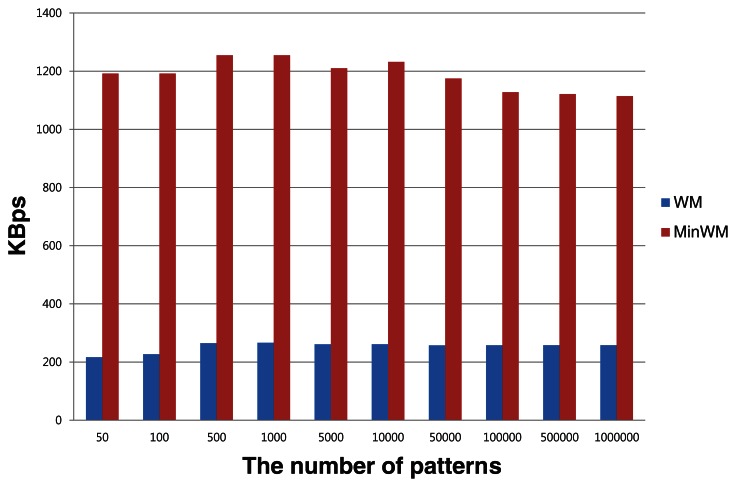
The average throughput of the two pattern sets in the base station.

Figure 8 shows the average throughput of the two pattern sets. In MinWM, the suffix and prefix matching steps are skipped by the bookmarks. MinWM only performs the entire pattern comparison using the pre-written bookmarks. By comparing the results, MinWM shows faster detection time of suspicious strings than the general WM algorithm. The figure indicates that MinWM is on average 4.76 times faster. The faster detection helps the base station to gather sensing information more efficiently. The improved detecting performance also helps to manage more sensor nodes. Considering that many sensor nodes are typically utilized for large-scale sensing activities, higher density sensing environments can possibly be provided by the increased capacity.

The performance improvement is caused by the skipping schemes of the MinWM framework. The entire suffix- and prefix-matching steps are skipped in the base station. Although the exact-matching step takes a large portion of the execution time, reducing the amount of memory access to shift characters decreases the execution time. Furthermore, large numbers of payloads are considered as regular sensing activities without detection by the base station. Table 2 lists the average number of patterns that are examined by the exact-matching step. Only 2.55% of the patterns is alerted by the sensor node, and the rest is regarded as safe. Compared with the RIDES approach that we selected, fewer packets are examined by the base station, although the rates for RIDES depend on the types of hash functions. The results in the Table 2 indicate that MinWM can reduce the incidence of entire matching execution. From the analyses, the MinWM framework executes intrusion detection tasks more efficiently and has the capability to protect large numbers of sensor nodes from malicious DoS attacks.

## 6. Conclusions

In this paper, the concept of a distributed WM algorithm called MinWM has been introduced to establish an NIDS for low-end sensor nodes. To solve the problem of constrained resources, the WM algorithm is divided into two parts; the sensor nodes execute smaller part and the base station executes the other. In addition, the sensor nodes use modified WM tables to reduce memory burden. MinWM outperforms the general WM and RIDES approaches in a number of evaluations. One possible explanation for these results is that the skipped shifting step occupies a large portion of the execution time. The base station is more efficient if it executes only the actual matching step. However, the evaluations were conducted for a limited range due to the restricted string pattern sets. MinWM may be required to design systematic evaluations with actually deployed multiple sensor nodes and activities. Nevertheless, the study is still valuable because the evaluations prove the efficiency of the proposed structures and provide the basis for distributed work to prevent malicious attacks. We expect that the MinWM framework can be implemented with the whole Snort signatures using a large scale distributed system with more nodes.

This study provides an example of implementing high resource demanding work such as multiple pattern-matching algorithms to constrained sensor devices. Making cooperative detection frameworks among the different sensor nodes is possible because MinWM is developed from a standard sensing framework. The MinWM framework can also be applied to any other compatible framework that consists of a large number of sensor nodes utilized for many practical fields. In addition, the automated intrusion defense agent can be implemented by enhancing the detection functions on the base station. To achieve these objectives, the study of effective communication frameworks among the sensors would be recommended to establish efficient network systems. This study provides a valuable resource for the future study of cooperative systems among a large number of sensor nodes and base stations.

## Figures and Tables

**Table 1.** Required resources to examine a single packet.

**# of Bookmarks**	**MinWM**		**RIDES**		**Speedup Ratio**

	**Time** (*ms*)	**Power (***μ****W****s*)	**Time** (*ms*)	**Power (***μ****W****s*)	
0	0.204	1.51	39.533	128.08	193.79
5	0.417	3.08	39.547	128.13	94.84
10	1.106	8.16	39.436	127.77	35.66
15	1.248	9.21	39.562	128.18	31.70
20	1.408	10.39	39.550	128.14	25.96

**Table 2.** The number of alerted patterns that require actual matching.

Patterns	50	100	500	1000	5000	10,000	50,000	100,000	500,000	1,000,000
MinWM										

Average Counts	1.16	2.69	12.93	24.70	124.43	253.43	1275.40	2527.52	12680.77	25375.59
Rates (%)	2.3200	2.6900	2.5860	2.4700	2.4886	2.5343	2.5508	2.5275	2.5362	2.5376

RIDES										

Average Counts	2.64	5.16	25.63	50.33	251.27	506.09	2513.65	5040.52	25194.83	50345.80
Rates (%)	5.2800	5.1600	5.1260	5.0330	5.0254	5.0609	5.0273	5.0405	5.0390	5.0346
